# The Effect of Two Health Education Packages on Nutritional Knowledge, Practices, and Physical Activity Levels Among School-Going Adolescents in Rishikesh: A Cluster Randomized Trial

**DOI:** 10.7759/cureus.63950

**Published:** 2024-07-06

**Authors:** Rupsha Mallick, Ranjeeta Kumari, Yogesh Bahurupi, Anjali M, Nisarg Aravindan, Meghna Singh

**Affiliations:** 1 Department of Community and Family Medicine, All India Institute of Medical Sciences, Rishikesh, IND; 2 Department of Family and Community Medicine, All India Institute of Medical Sciences, Rishikesh, IND

**Keywords:** health education, physical activity, food practice, nutrition knowledge, adolescents

## Abstract

Introduction

Adolescence is a critical period known for presenting specific challenges in disease treatment and health promotion. Studies have highlighted that increased nutritional awareness is associated with healthier eating habits, while regular physical activity aids in controlling and preventing non-communicable diseases. Equipping adolescents with health education in schools prepares them to adopt and maintain healthy lifestyles throughout their lives. To assess and compare the efficacy of health education packages targeting nutrition knowledge, practices, and physical activity levels, a cluster-randomized trial was conducted among school-going adolescents.

Methodology

Two distinct health education packages were developed for the two intervention groups. In group 1, a health talk supported by a flip chart was delivered, followed by pamphlet distribution. In contrast, in group 2, only information pamphlets were distributed. The study was conducted in government schools in Rishikesh, with four schools selected. Two schools were randomly allocated to each intervention arm. In each school, a questionnaire was administered to assess the students' nutrition knowledge, practices and physical activity levels. This was followed by the intervention, and the students were reassessed for the same parameters after two weeks.

Result

The pre-intervention and post-intervention comparisons within the same group- the mean scores for nutrition knowledge, food practice, sleep duration, and recreational screen time were comparable in the intervention group 1 (p>0.05). There was a decrease in the mean physical activity score and screen time for studies in intervention group 1, and these differences were statistically significant (p<0.05). The mean scores for nutrition knowledge, physical activity, sleep duration, and screen time for studies were comparable in the intervention group 2 (p>0.05). There was an increase in the mean food practice score and a decrease in recreational screen time in intervention group 2, and these differences were statistically significant (p<0.05). On comparing the groups with each other, it was found that Group 2 exhibited a significantly higher mean food practice score compared to group 1 post-intervention. While there was a statistically significant decrease in the mean physical activity score in intervention group 1, this group still had higher physical activity levels than group 2. Post-intervention, group 2 exhibited a higher screen time for studies compared to group 1. Both groups had comparable sleep durations at baseline and post-intervention, with intervention group 1's mean sleep duration falling within the recommended range set by the American Academy of Sleep Medicine. Regression analysis provided valuable insights into the relationship between baseline values of various variables and their post-intervention values, aiding in understanding the impact of the health education packages.

Conclusion

The findings emphasize the significance of incorporating nutrition and physical activity education into the curriculum of students.

## Introduction

Adolescence is increasingly recognized as a life period that poses specific challenges for treating disease and promoting health [1]. Adolescent-developed eating habits may be a factor in nutrition-related issues with long-term health effects [2]. One of the things that influence the nutritional status and eating behaviors of people, families, and communities is nutrition knowledge [3].

Studies have reported that higher levels of nutritional awareness were also linked to healthier eating habits, like eating more fruits and vegetables, having breakfast regularly and eating fewer harmful snacks and fast food [4].

The World Health Organization advises adolescents to engage in physical activity of moderate to vigorous intensity, mostly aerobic, for at least 60 minutes per day on average throughout the week. Limiting sedentary time, especially recreational screen time, is another recommendation. Regular exercise has been shown to help control and prevent non-communicable illnesses. It can also enhance mental health, quality of life, and well-being. It prevents hypertension and helps in maintaining a healthy weight [5]. Globally, in 2016, 81% of adolescents aged 11 to 17 years were not physically active enough [6].

The building blocks for future health can be established throughout the crucial period of adolescence. Adolescents who get health education in school are better prepared with the knowledge, attitudes, and abilities necessary to adopt and maintain healthy lifestyles throughout their lives [7].

The objectives of this study are: 1) to assess and compare the effectiveness of two different health education packages on nutrition knowledge, practices, and physical activity levels of school-going adolescents in Rishikesh; and 2) to develop two health education packages for the intervention.

## Materials and methods

This was a school-based cluster randomized trial. The study was conducted in government schools of Rishikesh, Uttarakhand. Permissions were obtained from school authorities in Vikaskhand Doiwala, and four co-educational schools were selected from six government schools. Two schools were randomly allocated under each intervention arm. A cluster random sampling technique was used. Random allocation of schools was done in each intervention arm via a serially numbered sealed opaque envelope system. The study was an open-label study conducted over a period of six months.

Inclusion and exclusion criteria

Students fulfilling the following criteria were included in the study: 1) only students studying in the 9th and 11th grades of the selected schools. Since one school had students enrolled till 10th grade only, therefore in order to meet the required sample size, available students enrolled in class 10 were included; 2) students who gave their assent and their parent's consent; and 3) students who were present on the day of baseline data collection. Students who were unwilling to participate or comprehend were excluded from the study.

Data collected has been used only for research purposes and will remain protected from disclosure outside of the research setting or to unauthorized persons. Before beginning the study, permission was taken from the Institutional Ethics Committee of All India Institute of Medical Sciences (AIIMS) Rishikesh, the Block Education Officer, and school principals. Informed written consent and assent were obtained from the parents/ guardian and adolescents, respectively. Clinical Trials Registry- India (CTRI) registration (registration number: CTRI/2023/01/048945) was done before beginning data collection. Permission to use the nutrition knowledge questionnaire was taken from the author. Questionnaires for food practice and physical activity were under open access.

Sample size

Sample size calculation for number of clusters using difference between means and standard deviation (SD) was done using WinPepi software (version 11.65), using the following parameters from a study conducted by Rao et al. [8] - significance level of 5%, power of 80%, ratio B:A of 1, SD in A 16.13, SD in B 12.3, difference of 9.11, intraclass coefficient (ICC) of 0.03 [9], and cluster size of 50.

Required sample

The number of clusters required was four (two clusters in each group, assuming similar cluster sizes and similar ICCs). Therefore, two schools, i.e., clusters, were taken in each arm. Based on a cluster size of 50 in each school, it was expected to recruit a minimum of 100 students in each arm. A random number of students from the eligible classes who were present on the day of data collection were enrolled to fulfill the required sample.

Study tools

To evaluate nutrition knowledge and food practices, questionnaires from previously conducted studies were used [10, 11]. To measure the physical activity level, the International Physical Activity Questionnaire (IPAQ) was used [12]. Additionally, sociodemographic and anthropometric details, sleep duration, and screen time were also recorded.

Development of health education materials

Health education was delivered via a flip chart and pamphlets. First, the information content of the material was finalized and then they were designed in a manner that would be interesting for school students. The main contents of the material included information about constituents of diet (carbohydrates, fats, proteins, vitamins, and minerals); information on calories, nutrients, nutritious dietary habits, and foods; the amount of various foods an adolescent must consume; WHO's recommendation on physical activity for adolescents; and information on junk food and inactivity, screen time and sleep duration.

Baseline data collection

Baseline data was obtained from the students with the help of a self-administered questionnaire before delivering the intervention.

Intervention

Intervention One in Group 1

A health talk was delivered with the help of a flip chart. After the health talk, a pamphlet containing the same information was distributed among all the students for sustained exposure to information.

Intervention 2 in Group 2

Intervention in group 2 (control arm) was delivered only by the distribution of the information pamphlets.

Follow-Up

The post-intervention data were obtained two weeks after the intervention using the same questionnaire that was used in the baseline.

Data analysis was done using Microsoft Excel (Microsoft, Redmond, Washington) and SPSS (version 25; IBM Inc., Armonk, New York).

## Results

**Figure 1:**
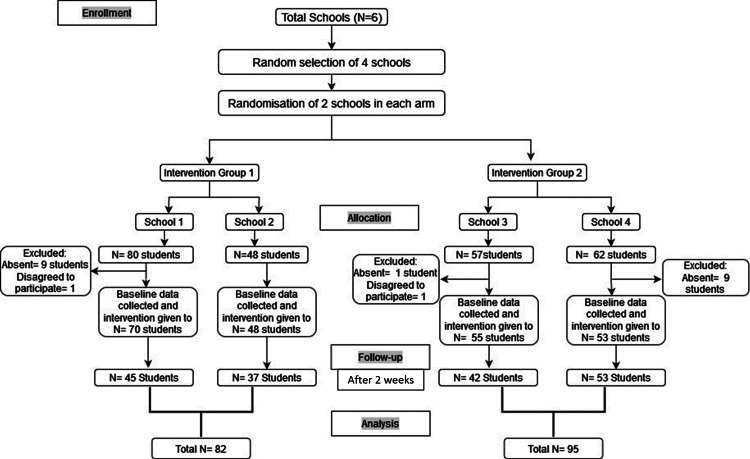
CONSORT diagram

The CONSORT diagram is shown in Figure 1.

Table 1 shows the comparison of the sociodemographic characteristics between the two groups. There was a statistically significant difference in the mean age of participants in the intervention group 1 (15.79±1.40) and intervention group 2 (16.60±1.00) groups (p<0.05). The groups were comparable in terms of gender, type of family, and number of siblings (p>0.05). There was a statistically significant difference seen in the class in which the participants were studying, socioeconomic status, occupation of mother, and BMI of the participants (p<0.05).

**Table 1:** Comparison of the sociodemographic characteristics between the two groups a - Mann-Whitney U test; b - Chi-squared test; * - denominator was 115 as three students did not tell the details

Variables		Intervention group 1, N=118		Intervention group 2, N=108		Test statistic, p-value
		N	%	N	%	
Age in years, mean (SD)		15.79 (1.40)		16.60 (1.00)		4271.0, <0.001^a^
Class	9	46	39.0	25	23.2	6.56, 0.010^b^
	10-11	72	61.0	83	76.9	
Gender	Male	37	31.4	26	24.1	1.48, 0.22^b^
	Female	81	68.6	82	75.9	
Type of family	Nuclear	70	59.3	78	72.2	4.51, 0.11^ b^
	Joint	21	17.8	11	10.2	
	Three-generation	27	22.9	19	17.6	
Socioeconomic status	Upper-middle and lower-middle	43	36.4	58	53.7	6.80, 0.009^ b^
	Upper-lower and lower-class	75	63.6	50	46.3	
Occupation of mother	Employed	50	42.4	24	22.2	10.40, 0.001^ b^
	Homemaker	68	57.6	84	77.8	
Number of Siblings	Siblings ≤2	30	26.1	27	25.0	0.04, 0.85^ b^
	Siblings >2	85	73.9*	81	75.0	
BMI	Underweight/ overweight	33	28.0	51	47.2	8.95, 0.003^b^
	Normal	85	72.0	57	52.8	

Table 2 shows the comparison of nutrition knowledge, food practice, and physical activity between the two groups at the baseline. Sleep duration and screen time for studies were comparable in the two groups (p>0.05). There was a statistically significant difference seen in nutrition knowledge, food practice, physical activity, and recreational screen time between the groups (p<0.05). Intervention group 2 had a higher nutrition knowledge mean score, lower food practice mean score, lower physical activity mean score, and higher mean recreational screen time. A smaller proportion of intervention group 1 students scored "good" for nutrition knowledge, and a higher proportion of students from intervention group 1 scored "high" for physical activity compared to intervention group 2.

**Table 2:** Comparison of the nutrition knowledge, food practice, and physical activity between the two groups at the baseline a - Mann-Whitney U test; b - Chi-squared test; MET - metabolic equivalent of task

Variables	Intervention group 1, N=118			Intervention group 2, N=108			Test statistic, p-value
	No.	%		No.	%		
Nutrition knowledge score (mean, SD)	8.42 (2.29)			9.20 (2.62)			5094.50, 0.009^a^
Nutrition knowledge category							
Low	58	49.1		38	35.2		6.28, 0.043^b^
Average	37	31.4		35	32.4		
Good	23	19.5		35	32.4		
Food practice score (mean, SD)	5.49 (1.64)			4.71 (1.86)			4924.00, 0.003^a^
Physical activity in total MET - minutes/week, mean (SD)	4945.16 (4833.88)			2469.29 (3980.50)			3724.00, <0.001^a^
Physical activity category							
Low	7		5.9	26		24.1	29.37, <0.001^b^
Moderate	49		41.5	59		54.6	
High	62		52.5	23		21.3	
Sleep duration in hours, (mean, SD)	7.87 (1.85)			7.73 (1.44)			6274.00, 0.84^a^
Recreational screen time in hours, (mean, SD)	1.84 (1.52)			2.47 (2.08)			5030.50, 0.02^a^
Screen time for studies in minutes, (mean, SD)	100.27 (65.36)			103.55 (82.64)			5994.50, 0.86^a^

Table 3 shows the comparison of the nutrition knowledge, food practice, and physical activity between the two groups after two weeks. The groups were comparable in terms of nutrition knowledge score and category, sleep duration, and recreational screen time (p>0.05). There was a statistically significant difference seen in food practice score, physical activity score, and category and screen time for studies between the two groups (p<0.05). The intervention group 2 had a higher mean food practice score and lower physical activity score. A higher proportion of students were in the "high" physical activity category from intervention group 1. The mean screen time for studies was lower in the intervention group 1.

**Table 3:** Comparison of the nutrition knowledge, food practice, and physical activity between the two groups after weeks weeks a - Mann-Whitney U test; b - Chi-squared test; MET - metabolic equivalent of task

Variables	Intervention group 1, N=82		Intervention group 2, N=95		Test statistic, p-value
	N	%	N	%	
Nutrition knowledge score (mean, SD)	8.83 (2.36)		9.45 (2.81)		3436.00, 0.17^a^
Nutrition knowledge category					
Low	36	43.9	33	34.7	1.629, 0.44^b^
Average	22	26.8	28	29.5	
Good	24	29.3	34	35.8	
Food practice score (mean, SD)	5.63 (1.54)		6.17 (1.76)		3177.00, 0.03^a^
Physical activity in total MET - minutes/week, mean (SD)	3748.21 (5182.08)		3128.47 (4416.42)		2817.50, 0.002^a^
Physical activity category					
Low	10	12.2	23	24.2	12.45, 0.002^b^
Moderate	22	26.8	39	41.1	
High	50	61.0	33	34.7	
Sleep duration in hours, (mean, SD)	8.27 (1.98)		7.62 (1.78)		3568.50, 0.31^a^
Recreational screen time in hours, (mean, SD)	2.23 (3.38)		2.04 (1.28)		3582.00, 0.41^a^
Screen time for studies in minutes, (mean, SD)	83.22 (59.55)		121.33 (108.89)		3216.50, 0.04^a^

Table 4 shows the pre-intervention and post-intervention comparisons within the same group. The mean scores for nutrition knowledge, food practice, sleep duration, and recreational screen time were comparable in intervention group 1 (p>0.05). There was a decrease in the mean physical activity score and screen time for studies in intervention group 1, and these differences were statistically significant (p<0.05). The mean scores for nutrition knowledge, physical activity, sleep duration, and screen time for studies were comparable in the intervention group 2 (p>0.05). There was an increase in the mean food practice score and a decrease in recreational screen time in intervention group 2, and these differences were statistically significant (p<0.05).

**Table 4:** Pre-intervention and post-intervention comparison of means of nutrition knowledge, food practices and physical activity, sleep duration, recreational screen time, and screen time for studies within groups a - Wilcoxon Signed Rank Test; MET - metabolic equivalent of task

Variable	Intervention Group 1		Test statistic, Pp-alue	Intervention Group 2		Test statistic, p-value
	Pre	Post		Pre	Post	
Nutrition knowledge score, (mean, SD)	8.49 (2.08)	8.83 (2.36)	-1.39, 0.16^ a^	9.08 (2.70)	9.45 (2.81)	-1.32, 0.19^a^
Food practice score, (mean, SD)	5.52 (1.64)	5.63 (1.54)	-0.51, 0.61^ a^	4.74 (1.86)	6.17 (1.76)	-6.00, <0.001^ a^
Physical activity in total MET - minutes/week, (mean, SD)	4945.16 (4833.88)	3748.21 (5182.08)	-1.12, 0.028^ a^	2469.29 (3980.50)	3128.47 (4416.42)	-2.45, 0.150^ a^
Sleep duration in hours, (mean, SD)	7.98 (1.86)	8.27 (1.98)	-1.41, 0.16^ a^	7.75 (1.32)	7.62 (1.78)	-0.31, 0.76^ a^
Recreational screen time in hours, (mean, SD)	1.81 (1.25)	2.23 (3.38)	-0.64, 0.52^ a^	2.55 (2.01)	2.00 (1.23)	-2.67, 0.004^ a^
Screen time for studies in minutes, (mean, SD)	99.42 (66.33)	83.22 (59.55)	-2.20, 0.03^ a^	102.92 (73.14)	122.59 (110.60)	-1.46, 0.15^ a^

Table 5 shows the linear regression analysis for the prediction of post-intervention nutrition knowledge score. The overall regression was statistically significant (R2=0.19, F value of the model =5.03, p=<0.001). It was found that baseline nutrition knowledge score significantly predicted post-intervention nutrition knowledge score. Participants with higher baseline nutrition knowledge had higher post-intervention nutrition knowledge scores.

**Table 5:** Linear regression analysis for prediction of post-intervention nutrition knowledge score SES - socioeconomic status; MET - metabolic equivalent of task

Predictors	Unstandardized coefficients	Standardized coefficients	Sig	95% CI for B	
	B	Beta		Lower	Upper
(Constant)	5.91		.09	-.93	12.75
Age	-0.08	-0.04	.72	-.52	.36
Gender	0.06	0.01	.89	-.76	.88
SES category	-0.26	-0.06	.40	-.85	.34
Group	0.44	0.08	.30	-.39	1.27
Baseline total nutrition knowledge	0.43	0.40	<0.001	.28	.59
Baseline total food practice	0.03	0.02	.80	-.18	.24
Baseline total MET score- minutes/week	4.333E-05	0.08	.32	.000	.000
Class category	0.32	0.06	.58	-.81	1.45
Model summary: R=0.44, adjusted R Square=0.16, F value of the model=5.03, p-value<0.001					

Table 6 shows the linear regression analysis for the prediction of post-intervention food practice scores. The overall regression was statistically significant (R2=0.20, F value of the model =5.39, p=<0.001). It was found that baseline food practice score and group significantly predicted post-intervention food practice score. Participants with higher levels of baseline food practice scores and being in the intervention arm two were associated with higher scores on post-intervention food practice scores.

**Table 6:** Linear regression analysis for prediction of post-intervention food practice score SES - socioeconomic status; MET - metabolic equivalent of task

Predictors	Unstandardized coefficients	Standardized coefficients	Sig	95% CI for B	
	B	Beta		Lower	Upper
(Constant)	2.48		0.26	-1.87	6.82
Age	-0.04	-0.03	0.77	-0.32	0.24
Gender	-0.02	-0.01	0.93	-0.54	0.50
SES category	0.08	0.03	0.67	-0.30	0.46
Group	0.95	0.28	0.001	0.42	1.48
Baseline total nutrition knowledge	0.05	0.07	0.31	-0.05	0.15
Baseline total food practice	0.38	0.41	<0.001	0.24	0.51
Baseline total MET score- minutes/week	3.286E-5	0.09	0.23	0.000	0.000
Class category	-0.05	-0.01	0.90	-0.76	0.67
Model Summary: R=0.45, Adjusted R Square=0.17, F value of the model=5.39, p-value: <0.001					

Table 7 shows the linear regression analysis for the prediction of post-intervention physical activity level score (expressed as metabolic equivalent of task (MET) score). The overall regression was statistically significant (R2=0.28, F value of the model =8.21, p=<0.001). It was found that gender and baseline physical activity level scores significantly predicted post-intervention physical activity level scores. Participants who were female were associated with lower post-intervention physical activity level scores. Participants having higher baseline physical activity level scorea had higher post-intervention physical activity level scorea.

**Table 7:** Linear regression analysis for prediction of post-intervention physical activity level score (expressed as MET score) SES - socioeconomic status; MET - metabolic equivalent of task

Predictors	Unstandardized coefficients	Standardized coefficients	Sig	95% CI for B	
	B	Beta		Lower	Upper
(Constant)	2506.65		0.69	-9949.85	14963.14
Age	508.46	0.13	0.21	-293.64	1310.55
Gender	-3202.31	-0.29	<0.001	-4697.01	-1707.62
SES category	74.70	0.01	0.89	-1011.00	1160.39
Group	-1124.02	-0.11	0.14	-2637.27	389.22
Baseline total nutrition knowledge	-92.51	-0.05	0.51	-370.27	185.26
Baseline total food practice	80.23	0.03	0.68	-305.23	465.70
Baseline total MET score- minutes/week	0.38	0.34	<0.001	0.22	0.53
Class category	-205.72	-0.02	0.84	-2261.83	1850.38
Model summary: R=0.53, Adjusted R Square=0.25, F value of the model=8.21, p-value<0.001					

Table 8 shows the linear regression analysis for prediction of post-intervention sleep duration. The overall regression was statistically significant (R2=0.30, F value of the model=9.87, p=<0.001). It was found that group and baseline sleep duration significantly predicted post-intervention sleep duration. Participants who were from intervention group 2 were associated with lower post-intervention sleep duration. Participants with higher baseline sleep duration had higher post-intervention sleep duration.

**Table 8:** Linear regression analysis for prediction of post-intervention sleep duration SES - socioeconomic status

Predictors	Unstandardized coefficients	Standardized coefficients	Sig	95% CI for B	
	B	Beta		Lower	Upper
(Constant)	5.31		0.03	0.38	10.23
Age	-0.20	-0.13	0.21	-0.51	0.11
Gender	0.27	0.06	0.35	-0.30	0.84
SES category	0.11	0.04	0.60	-0.29	0.51
Group	-0.58	-0.15	0.04	-1.15	-0.02
Class category	0.76	0.18	0.06	-0.02	1.54
Baseline sleep duration (hours)	0.55	0.46	0.00	0.39	0.71
Baseline recreation screen time (hours)	0.12	0.11	0.13	-0.03	0.27
Model summary: R=0.54, adjusted R Square=0.27, F value of the model=9.87, p-value<0.001					

Table 9 shows the linear regression analysis for prediction of post-intervention recreation screen time. The overall regression was statistically not significant (R2=0.06, F value of the model =1.38, p=0.22). It was found that baseline recreational screen time significantly predicted post-intervention recreational screen time. Participants having higher baseline recreational screen time had higher post-intervention recreational screen time.

**Table 9:** Linear regression analysis for prediction of post-intervention recreation screen time SES - socioeconomic status

Predictors	Unstandardized coefficients	Standardized coefficients	Sig	95% CI for B	
	B	Beta		Lower	Upper
(Constant)	-0.07		0.98	-7.46	7.32
Age	0.20	0.10	0.39	-0.26	0.66
Gender	0.07	0.01	0.87	-0.79	0.93
SES category	-0.28	-0.07	0.36	-0.88	0.32
Group	-0.73	-0.15	0.09	-1.58	0.12
Class category	0.25	0.05	0.67	-0.92	1.42
Baseline sleep duration (hours)	0.00	0.00	0.97	-0.25	0.24
Baseline recreation screen time (hours)	0.23	0.16	0.04	0.01	0.46
Model summary: R=0.24, adjusted R Square=0.02, F value of the model=1.38, p-value=0.22					

## Discussion

A previously conducted study indicated a significant improvement in the nutrition knowledge levels of adolescent girls after a traditional method of intervention, which was intervention through class teachers by using print media [7]. At follow-up, this present study showed that there was an increase in the nutrition knowledge of the students, although not statistically significant. The results of this study indicate that despite the lack of statistical significance, the intervention could be helpful for certain students.

In this study, group 2 showed a significantly higher mean food practice score than group 1 at the follow-up. On comparison within the groups, group 1 showed an improvement that was not statistically significant, and group 2 showed a statistically significant improvement.

At the follow-up, though there was a statistically significant drop in the mean physical activity score seen in intervention group 1, group 1 had a statistically significantly higher physical activity compared to group 2. Numerous school-based physical activity initiatives have shown favorable results, according to a previously completed systematic review [13].

There was a statistically significant decrease in mean recreational screen time observed in group 2. The initial screen time for studies was similar in both groups at the baseline. However, at the follow-up assessment, a statistically significant difference was observed between the two groups, indicating that intervention group 2 had higher screen time for studies compared to intervention group 1. Given the prevalent use of electronic media and audio-visual aids in modern-day studies, it is common for individuals to have a high screen time. However, it is crucial to make efforts to reduce screen usage for non-academic purposes, such as recreation. At both the baseline and post-intervention assessments, the sleep duration between the two groups was found to be comparable. The mean sleep duration in intervention group 1 fell within the recommended range set by the American Academy of Sleep Medicine. This suggests that the intervention had a positive impact on promoting adequate sleep duration in participants belonging to this group.

The regression analysis has provided valuable insights into the relationship between the baseline values of various variables and their post-intervention values. The findings emphasize the significance of incorporating nutrition education and physical activity education into the curriculum of students.

## Conclusions

This study has shed light on the effectiveness of health education packages in improving the nutrition knowledge, food practices, physical activity levels, sleep duration and screen time habits of school-going adolescents. While all the results were not statistically significant, they indicate that the interventions have the potential to make a positive impact on students' health-related behaviours.

By integrating health education throughout the educational journey, students are more likely to develop and maintain healthy habits that can improve their overall well-being. Sustained release of information and repeated interventions may be necessary to achieve meaningful and long-term behaviour change.
